# Relevance of frozen sections and serum markers in invasive squamous cell carcinoma arising from ovarian mature cystic teratoma: two case reports

**DOI:** 10.1186/s13256-015-0783-5

**Published:** 2016-01-22

**Authors:** Yuki Tazo, Yoshihiro Yoshimura, Takashi Shoda, Noriyuki Kyushima, Takemichi Okada, Hitoshi Yamazaki

**Affiliations:** 1 Department of Gynecology and Obstetrics, Kitasato University, 1-15-1 Kitasato, Minami, Sagamihara, Kanagawa Japan; 2 Department of Radiology, Kitasato University, 1-15-1 Kitasato, Minami, Sagamihara, Kanagawa Japan; 3 Department of Pathology, Kitasato University, 1-15-1 Kitasato, Minami, Sagamihara, Kanagawa Japan

**Keywords:** Squamous cell carcinoma, Mature cystic teratoma, Ovary, Immunohistochemical analysis

## Abstract

**Background:**

Ovarian mature cystic teratoma (MCT) is a common neoplasm in women. While malignant transformation of MCT is relatively rare, squamous cell carcinoma is the most frequent malignant neoplasm arising from MCT. Some tumor markers have been reported to be useful for prediction of MCT malignant transformation prior to operation. However, widely accepted use of these markers remains to be established. In the present study, we report the usefulness of frozen section assessment during operation, as well as preoperative measurement of tumor marker levels.

**Case presentation:**

We present two cases of squamous cell carcinoma arising from ovarian MCT. The first case was a 45-year-old Asian woman referred to our hospital after her periodical company medical checkup, due to possible ovarian tumor. Image analysis suggested a dermoid cyst, and left salpingo-oophorectomy was performed. Because the cyst was histologically diagnosed as an invasive squamous cell carcinoma arising from an MCT, our patient underwent an additional preventative operation. The TNM classification and FIGO stage were T1aNXM0 and Ia, respectively. The second case was a 53 -year-old Asian woman who visited our hospital due to complaints of abdominal pain and urinary retention. Image analysis and laboratory data showing high serum levels of SCC antigen (normal range: < 1.5 ng/mL) and CA19-9 (normal range: < 37 U/mL), which strongly suggested malignant transformation of MCT. Frozen sections obtained during the operation were histologically analyzed to confirm malignancy, and our patient underwent an additional operation. The TNM classification and FIGO stage were T1aNXM0 and Ia, respectively.

**Conclusions:**

We report the usefulness of frozen section assessment during operation, as well as preoperative measurement of tumor marker levels.

## Background

Ovarian mature cystic teratoma (MCT) is a common neoplasm in women. While malignant transformation of MCT is relatively rare [1, 2], squamous cell carcinoma (SCC) is the most frequent malignant neoplasm arising from MCT [3, 4]. Preoperative levels of tumor markers, such as SCC antigen, are useful for identifying the presence of SCC arising from MCT [5, 6]. However, there have been previously reported cases of other types of malignancies arising from MCT, such as neuroendocrine carcinoma [7–11], which are unable to be identified by preoperative evaluation of SCC antigen. The prognoses of patients with such secondary malignancies depend on their International Federation of Gynecology and Obstetrics (FIGO) stage [4, 7, 12, 13]. To improve patient quality of life, we investigated the usefulness of frozen section assessment during operation for confirmation of MCT malignant transformation.

## Case presentation

### Case 1

**Fig. 1:**
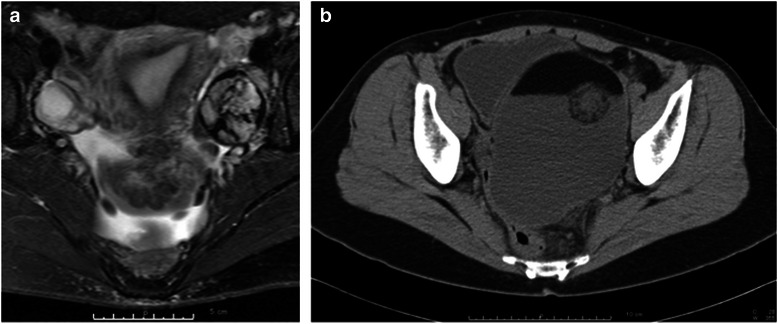
Image analysis. **a** Gadolinium-enhanced T1-weighted magnetic resonance imaging scan of the left ovary shows a cystic lesion, approximately three-fourths of which is occupied by an irregularly enhanced mass. **b** An enhanced computed tomography scan shows a large intra-abdominal cystic lesion occupying the pelvic cavity. Focal intracystic irregular enhancement is noted

**Fig. 2:**
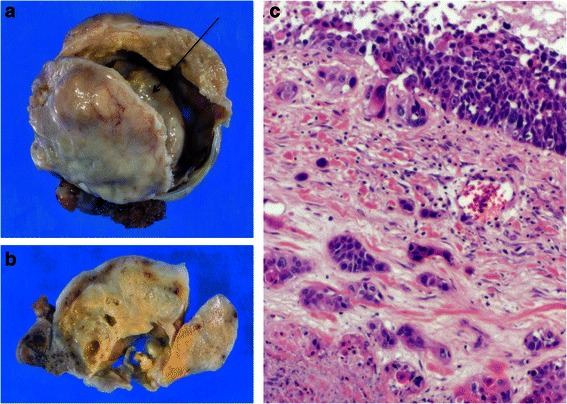
Gross findings and microscopic findings of case 1. **a** The inner surface of the cyst wall is irregularly thickened. A firm nodule is located within the cyst (*arrow*). **b** The cut surface of the cyst contains yellowish-gray, firm areas, and a few cystic cavities are embedded within. **c** A hematoxylin and eosin-stained section shows scattered islands of invasive squamous cell carcinoma (× 200)

A 45-year-old Asian woman was referred to the gynecology and obstetrics department of our hospital after a periodical company medical checkup, due to possible ovarian tumor. Ultrasound and magnetic resonance imaging (MRI) scans revealed an intra-abdominal cyst, approximately 44 mm in diameter. Periodical follow-up imaging later revealed a hairball in the cyst, leading to diagnosis of dermoid cyst. Five years after initial presentation, the size of the cyst measured 59 x 59 mm (Fig. 1a), and serum levels of SCC antigen and cancer antigen (CA)125 (normal range: < 35 U/mL) were 1.4 ng/mL and 10.8 U/mL, respectively. Follow-up biochemical analysis revealed that the level of SCC antigen gradually increased over a period of 6 months, to 4.8 ng/mL. Our patient subsequently underwent left salpingo-oophorectomy due to suspicion of malignant transformation of the ovarian cyst. At laparotomy, bloody ascites in the peritoneal cavity were observed, and the cytological analysis of the ascites fluid during the operation revealed no malignant cells. On gross examination, the resected ovarian cyst contained a number of mature hair shafts intermingled with abundant atheromic material. Frozen section analysis was not performed because the cyst was macroscopically diagnosed as a dermoid cyst. After fixation by formaldehyde, the cyst walls were observed to be thickened, with protruding, irregular nodules partially filling the cyst (Fig. 2a). The cut surface of the thickened cyst wall was yellowish and solid (Fig. 2b). Histologically, the multilocular cystic space was lined by mature squamous epithelium, and many carcinomatous foci were observed. Invasive growth of SCC was pronounced in the cyst wall (Fig. 2c). These findings led to a diagnosis of invasive SCC (well-differentiated SCC) arising from MCT. No vascular invasion was identified. One month after the first operation, our patient underwent an additional preventive operation (a simple abdominal hysterectomy and right salpingo-oophorectomy). Lymphadenectomy and omentectomy were not performed due to our patient’s wish for limited surgery. Pathological analysis revealed no residual carcinoma in the uterus, right ovary, or right fallopian tube. The TNM classification and FIGO stage were T1aNXM0 and Ia, respectively. Our patient was started on paclitaxel and carboplatin chemotherapy postoperatively; 22 months later, she remained free of any clinically identifiable recurrence.

### Case 2

**Fig. 3:**
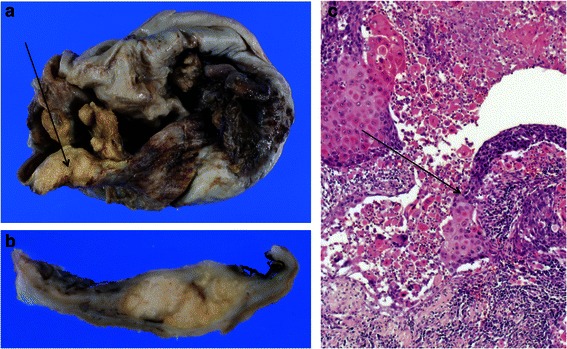
Gross findings and microscopic findings of case 2. **a** The wall of the cyst is thickened (*arrow*). **b** The cut surface of the cyst shows nodular protrusions. **c** A hematoxylin and eosin-stained section of the nodular area shows the boundary between the dysplastic keratinocytes lining the cyst internally, and the invasive squamous cell carcinoma (*arrow*) (× 100)

A 53-year-old Japanese woman presented at the gynecology and obstetrics department of our hospital due to complaint of abdominal pain and urinary retention. A computed tomography (CT) scan revealed an intra-abdominal cyst measuring 130 x 135 mm, and an MRI scan showed focal thickening of the cyst wall. Laboratory analysis revealed high serum levels of SCC antigen (6.2 ng/mL) and CA19-9 (344 U/mL). The possibility of malignant transformation of the dermoid cyst was thought to be low, and systemic CT suggested no distant metastasis. Forty-two days after initial presentation, our patient underwent surgery. At laparotomy, yellowish ascites was observed in the peritoneal cavity, and gross examination revealed that the cyst wall was thickened and attached to the left fallopian tube, small intestines, posterior wall of the uterus, and retroperitoneum. Frozen sections of the thickened wall of the cyst were prepared and histologically analyzed, revealing invasive SCC in the cyst wall. Cytological analysis of the ascites fluid during the operation revealed no malignant cells. Thus, malignant transformation was diagnosed, and our patient underwent a total abdominal hysterectomy, bilateral salpingo-oophorectomy, and omentectomy. However, lymphadenectomy was not performed due to the firm attachment of the cyst wall to the surrounding lymph nodes. Focal and irregular thickening of the cyst wall was observed after fixation by formaldehyde (Fig. 3a). The cut surface of the thickened cyst wall appeared yellowish and solid, and was connected directly to the inner surface of the cyst (Fig. 3b). Histological analysis revealed a unilocular cystic space lined by mature squamous epithelium. Malignant transformation was identified in the epithelial lining (Fig. 3c). These findings led to a diagnosis of SCC (well-differentiated SCC) arising from MCT. No definite vascular invasion was identified. The TNM classification and FIGO stage were T1aNXM0 and Ia, respectively. Twenty-one months postoperatively, our patient remained free of any clinically identifiable recurrence.

## Discussion

The etiology of malignant transformation in MCT has not yet been elucidated. Approximately 75 % of malignant transformations are categorized as SCCs [14]. Iwasa *et al*. speculated that SCC may arise from metaplastic squamous epithelium located within the MCT [1]. It has also been suggested that long-term presence of MCT without removal may be associated with malignant transformation [15]. We have experienced another case of MCT with focal nodular hyperplasia of the squamous epithelium lining, similar in appearance to ‘seborrheic keratosis’ and with a preoperative SCC serum level of over 1.0 ng/mL (data not shown). Furthermore, the internal lining of the squamous epithelium of the mature cystic teratoma may also have potential for growth.

Some tumor markers have been reported to be useful for MCT characterization and prediction of malignant transformation [16]. Hackethal *et al*. analyzed the age, presence of tumor markers, symptoms, and FIGO stages of 277 cases described in the literature from 1978 to 2007 [2]. Kataoka *et al*. reported that the level of CA125 and CA19-9, but not SCC antigen, was significantly correlated with MCT tumor diameter [16]. The significance of high SCC antigen serum levels has been reported in several cases of malignant MCT transformation [5, 6, 17, 18], with a SCC cutoff level of 2.0 ng/mL [6, 17, 18], and Mori *et al*. utilized a SCC antigen cutoff level of 2.5 ng/mL in patients aged over 40 years old [5]. Furthermore, Kikkawa *et al*. demonstrated that patient age and ovarian tumor size are important factors in predicting SCC arising from ovarian MCT [19]. Choi *et al*. reported an average SCC antigen level of 1.5 ng/mL in patients with FIGO stage Ia tumors [20]. Futagami *et al*. assessed malignant transformation risk by tumor size and marker levels [21]. Suzuki *et al*. proposed the use of a combination of two serum markers, macrophage colony-stimulating factor (M-CSF) and SCC antigen, to preoperatively diagnose SCC arising from ovarian MCT [18].

Although there have been previous studies assessing the association between clinical findings and malignant transformation of MCT [3, 14], specific symptoms for malignant transformation remain to be identified. The present results suggested the usefulness of analyzing frozen sections during operation to identify malignant transformation of MCT. Furthermore, this technique provides the opportunity to detect malignancies other than SCC, which may also arise from ovarian MCT. If preoperative imaging reveals unnatural nodular areas within the cyst wall, histological analysis of frozen sections during the initial operation may avoid the need for a secondary operation for complete removal of the malignancy. Although prognosis depends on the FIGO stage [4, 7, 12, 13] and malignancy may not be identified in all cases [22], avoiding a secondary operation has been demonstrated to increase the patient’s quality of life.

In summary, we report two cases of SCC arising from ovarian MCT. We emphasize the usefulness of frozen section assessment during operation, as well as preoperative measurement of tumor marker levels. These methods facilitated detection of both SCC and other malignancies arising from MCT.

## Conclusions

Malignant transformation of mature cystic teratoma is rare. However, such transformation is difficult to identify prior to operation, if serum levels of tumor markers are near the cutoff level. The present results suggested the usefulness of frozen section assessment during operation, which may aid in increasing patient quality of life.

## Consent

Written informed consent was obtained from the patients for publication of both the case reports and any accompanying images. Copies of written consent are available for review by the Editor-in-Chief of this journal.

## Abbreviations

CA: Cancer antigen
CT: Computed tomography
FIGO: International Federation of Gynecology and Obstetrics
M-CSF: Macrophage-colony stimulating factor
MCT: Mature cystic teratoma
MRI: Magnetic resonance imaging
SCC: Squamous cell carcinoma
